# Interpretation and use of caloric testing

**DOI:** 10.1016/S1808-8694(15)30580-2

**Published:** 2015-10-19

**Authors:** Denise Utsch Gonçalves, Lilian Felipe, Tânia Mara Assis Lima

**Affiliations:** 1 MD. PhD. Otorhinolaryngologist, Adjunct Professor – Department of Ophthalmology, Otolaryngology and Speech and Hearing Therapy – University of Minas Gerais Medical School. Coordinator of the Vertigo ward – ENT Department of the University of Minas Gerais Medical School - FM-UFMG.; 2 Speech and hearing therapy – FM-UFMG. MSc student in Infectology and Tropical Medicine of the UFMG.; 3 MD. PhD. Otorhinolaryngologist, Adjunct Professor – Department of Ophthalmology, Otolaryngology and Speech and Hearing Therapy – University of Minas Gerais Medical School. Head of the University Hospital - University of Minas Gerais Medical School - FM-UFMG. University of Minas Gerais Medical School – Graduate Program in Infectology and Tropical Medicine.

**Keywords:** electronystagmography, interpretation, nystagmus, caloric testing, vestibular system

## Abstract

Caloric testing is an otoneurologic evaluation of the status of the vestibular-ocular reflex; it allows an evaluation of each labyrinth separately. The main aspects on the use and interpretation of caloric testing are reviewed.

**Method:**

A systematic review of papers published in the past one hundred years on caloric testing was undertaken. The inclusion criteria were: cross-sectional, longitudinal, original articles, reviews and meta-analyses. Reviews of patient charts, case reports and editorials were excluded. The key-words were: caloric testing, nystagmus, vestibular system, directional preponderance, labyrinth predominance, monothermal caloric test, ice water caloric testing, Bell´s phenomenon. The databases were: COCHRAINE, MEDLINE, LILACS, CAPES.

**Results:**

Ninety-three of 818 abstracts fulfilled the inclusion criteria. After reading these articles, 55 were selected for this study. These papers discussed the basics of caloric testing, the types of stimulation, the interpretation of results, variables, artifacts, and the monothermal and ice water caloric test.

**Final comments:**

Caloric testing reference values may vary according to each unit; the the cutoff point is defined based on local studies. Attention to the technique is essential to maximize test sensitivity.

## INTRODUCTION

Caloric testing assesses and records the function of each labyrinth separately, making it possible to define which side is compromised.^1^, ^2^ The caloric response is connected with the central nervous system, which is important in differentiating between central and peripheral vestibular diseases.

Caloric testing deserves to be highlighted within the context of otoneurological test batteries, which justifies this review of the literature.

Peculiarities about the semiotechnique should be well known when performing the test to avoid errors in the interpretation of results.

Furthermore, controversies seem to exist in the interpretation of caloric testing. For instance, directional preponderance and labyrinthic predominance reference values may vary among units, as may the clinical meaning of directional predominance and the advantages/disadvantages of air and water irrigation.

This systematic review aimed to describe the main aspects about the method, the interpretation and the clinical usefulness of caloric testing.

## METHODS

The bibliographic search included a review of strategic points for commanding the test technique, as follows:

1) air or water stimulation caloric testing;
2) monothermal caloric testing;
3) ice water caloric testing;
4) directional preponderance and labyrinthic predominance: concepts, reference values and associated diseases;
5) areflexia, hyporeflexia and hypereflexia: concepts, reference values and associated diseases;
6) variables and artifacts that affect the caloric response: lighting, temperature, habituation, anxiety, status of the tympanic membrane, use of drugs, blinking of the eyes, Bell's phenomenon.

The databases that we investigated were: CENTRAL (The Cochrane Controlled Trials Register), MEDLINE (Medical Literature, Analysis and Retrieval System on Line), LILACS (Latin American Health Science Literature - Literatura Latino Americana de Ciências da Saúde) and periodicals CAPES (Coordination of Higher Level Staff Training - Coordenação de Aperfeiçoamento de Pessoal de Nível Superior) for papers published between December 1905 and January 2006. The keywords used in the survey were: caloric testing, nystagmus, vestibular system, directional preponderance, vestibular weakness, monothermal caloric testing, abnormal reflex, ice water caloric test and Bell's phenomenon and their equivalents in Portuguese or Spanish.

Cross-sectional studies, prospective and retrospective longitudinal studies, review articles and meta-analyses were included. Reviews of patient charts, case reports and editorials were excluded.

## RESULTS

In these databases, 818 abstracts were found using the research keywords. Of these, 93 abstracts were selected. Reading of the complete papers resulted in the selection of 55 papers. In this phase, selection was based on the alignment between the definition of the topic and the aims of this paper. Those papers that did not deal with the method and interpretation of caloric testing were excluded from this review.

## DISCUSSION

### Basics of caloric testing

Caloric testing is based on the principle of generating thermal variation within the external auditory canal; by changing the temperature of the middle ear, this thermal variation changes the density of endolymph within the lateral semicircular canal, producing convection currents that stimulate the sensorial cells located in the ampullary crest. The patient is placed in dorsal decubitus at 30º relative to the horizontal plane. This position places the lateral canal vertically, as a liquid column, and places the ampullary crest superiorly.^1^, ^2^, ^3^

An upward change in the middle ear temperature above the bodily temperature causes the endolymph to move upwards, generating an endolymphatic current within the canal towards the ampulla. If the stimulus temperature is lower than the bodily temperature, there is the opposite movement, which generates an ampullary current towards the canal, away from the ampulla. The action of these convection currents on the ampullary crest alters the action potential of this sensory receptor, stimulating or inhibiting these currents. Stimulation initiates the vestibuloocular reflex (VOR), a simple reflex arc from the vestibular nucleus to the oculomotor nuclei, which generates the vestibular nystagmus. The nystagmographic response is evaluated and compared with a normal standard. Caloric testing does not assess the function of the sacculus or the utricle of the vertical canals.

Caloric stimulation may be done with water or air; it generates an endolymphatic current within the stimulated lateral canal, analogous to a 0.003 Hz angular movement.^4^ Knowing that the semicircular canals respond more efficiently to angular movements at 1 to 6Hz, it may be concluded that caloric testing assess the labyrinth in a non-physiological frequency.^4^, ^5^

### Stimulation: water or air

Barany^6^ described water caloric stimulation, and Fitzgerald and Hallpike7 established its standard protocol. The ear is water-irrigated for 40 seconds at temperatures of 44°C and 30°C, 7°C over and 7°C below the bodily temperature, which generates the endolymphatic current.^4^ Water stimulation induces more robust caloric responses and causes less variability among individuals compared to air stimulation.^8^, ^9^ On the other hand, water stimulation produces more frequent neurovegetative reactions compared to air stimulation.^10^

In air stimulation, an 8 l/min air current at 50°C and 24°C (13°C above and 13°C below body temperature) is applied for 60 seconds; this generates an endolymphatic current similar to that generated by water at 44°C and 30°C.^9^, ^11^ As air is not a good heat conductor, changes in the stimulation depth within the external auditory canal may reduce the slow phase of nystagmus by 20% to 40%.^8^ This technique, therefore, requires more technical expertise compared to water caloric stimulation.^8^, ^9^ Other stimulation standards have been used, such as: 45.5°C and 27.5°C during 100 seconds at 13 l/min air flow,8 or 18°C and 42°C during 80 seconds at 7 to 8 l/min air flow.^12^

### Interpretation

The principle underlying caloric stimulation is that normal labyrinths tend to respond symmetrically and measurably within a previously known normal range. An asymmetric response is related with current or past conditions.^2^ An absent or decreased response indicates peripheral vestibular dysfunction.^2^, ^4^ In this context, the interpretation of caloric testing should compare both sides as well as absolute values.

The comparative analysis seeks asymmetries in the caloric response, and focuses on analyzing labyrinthic paresis (LP) and directional predominance (DP).^2^ The analysis of absolute values evaluates whether those values are above or below a normal range. This gains importance if LP and DP are within normal limits but absolute values are out of the normal range. In absolute values, hyperreflexia is defined as nystagmographic responses higher than expected, hyporreflexia as responses lower than expected, and arreflexia as the absence of a caloric response.^1^, ^2^, ^3^

### Labyrinthic predominance

Jongkees^13^ published a formula for calculating LP (lost or decreased function of a labyrinth compared to the contralateral labyrinth) when comparing peripheral vestibular function. This author used water stimulation (250 ml per minute flow at 30°C and 44°C) and established that LP was a difference over 20% in the nystagmographic response between both sides; the worse labyrinth was that with the most deficient caloric response.^1^, ^2^, ^13^

Currently, the normal standard for defining vestibular paresis varies among different ENT units; values from 20% a 33%, which depend on internal normalization, have been described.^12^, ^13^, ^14^ In Brazil the terms labyrinthic paresis or weakness are uncommon; there is a preference for naming the best-responding labyrinth, using the denomination labyrinthic predominance. The end result does not logically change; rather, it depends only on how the process is viewed.

The most common causes of unilateral vestibular dysfunction are eighth cranial pair tumors,15 vestibular neuronitis,^16^ and Ménière's disease.^17^ Unilateral dysfunction may also occur in migraine and in cerebrovascular diseases.^19^

### Directional preponderance

Jongkees^13^ first defined DP as a trend for an increased intensity of nystagmus towards a certain direction compared with the other. As with LP, normal DP values vary among ENT units, ranging from 22% to 33%.^12^, ^13^, ^14^

The clinical meaning of DP is controversial. DP is commonly seen in patients presenting spontaneous nystagmus, occurring towards the same direction.20 It may also be observed in central or peripheral vestibular diseases or in injuries of the cortex.^7^, ^21^, ^22^, ^23^ DP may also be seen in normal subjects.^21^ Given such variability, DP does not always correlate with disease of the vestibular system, and has no value for locating the site of injuries.^22^, ^23^

Contralateral DP is frequently observed in unilateral peripheral deficit syndromes.^21^, ^22^, ^23^ DP may persist towards the opposite direction of the side involved, after the acute phase has regressed and spontaneous nystagmus has disappeared.^21^ Young unilateral peripheral deficit syndrome patients, in whom vision and proprioception are preserved, present temporary contralateral to the lesion DP during the vestibular compensation process; in these patients, DP tends to disappear as soon as compensation is complete.^21^, ^22^ This does not occur in elderly patients or those presenting limits for complete compensation; in these groups, contralateral to the lesion DP may be permanent.^22^ DP has been reported in Ménière's disease, although it has no use for establishing the side of the lesion.^17^, ^24^

DP has been observed in central diseases, such as involvement of eighth cranial nerve branches, of the brainstem and the cortex.^23^

### Hyperreflexia

Hyperreflexia may be associated with central or peripheral vestibular diseases; it is the situation in which caloric induced nystagmus exceeds the normal range. Some researchers have established that hyperreflexia is a nystagmographic response over 40º/seg to 80º/seg.^25^

In peripheral vestibular disease, hyperreflexia may be seen in the contralateral labyrinth to that with a deficient response.^22^, ^26^

Bilateral hyperreflexia may be observed in central vestibular diseases. In a state of normalcy, the cerebellar flocculus inhibits vestibular nucleus neurons, thus inhibiting the VOR.^23^, ^26^ Injury to this region affects this inhibitory function, increasing the excitatory state of the vestibular nucleus, resulting in bilateral hyperreflexia. This mechanisms explains bilateral hyperreflexia commonly seen in multiple sclerosis patients.^27^, ^28^ Bilateral hyperreflexia of no apparent cause - such as in anxiety or upon ingesting psychoactive drugs - added to fewer neurovegetative symptoms than expected for the observed hyperreflexia, may indicate central injury.^26^, ^27^, ^28^

Otological alterations unrelated to the peripheral or central labyrinthic lesion might generate hyperreflexia. The most common are those that favor transference of the caloric stimulus to the inner ear, such as mastoidectomy or tympanic membrane perforation/atrophy/retraction. Anxiety of patients is a further cause of hyperreflexia.^26^, ^29^ In fact, fear of caloric testing appears to be one of the most common causes of hyperreflexia.^26^, ^30^

### Hyporreflexia

In defining bilateral vestibular function deficits, some authors have suggested that patients should present a combination of caloric responses under 11°/seg upon bilateral warm irrigation, and 6°/seg for bilateral cold stimulation.^31^ In these cases, LP and DP would be normal, as there is no functional asymmetry. It would not, however, indicate normal vestibular function. Bilaterally decreased caloric responses may be seen in patients using drugs that depress labyrinthic function, such as cinnarizine and flunarizine.^32^ Causes of persistent hyporreflexia may be associated with ototoxicity, in which the caloric response ranges from hyporreflexia to arreflexia according to time and exposure dose.^33^, ^34^ Other causes of hyporreflexia are systemic infections, such as congenital or acquired syphilis,^35^ central nervous system diseases, such as supratentorial tumors,^36^ benign intracranial hypertension,^37^ and Friedreich's ataxia, a progressive hereditary neurodegenerative disease affecting mostly the spinal cord and the cervicomedullar junction.^38^ Metabolic diseases that may cause hyporreflexia include Wernicke-Korsakoff's encephalopathy, which is associated with thiamine deficiency in chronic alcoholism.^37^ Cogan's syndrome, a rare disease that affects the cochlear spiral ligament, causing sensorineural hearing loss, tinnitus and interstitial keratitis, may also cause bilateral hyporreflexia.^40^

### Arreflexia

Absence of a response in caloric testing characterizes arreflexia. Labyrinthic diseases causing arreflexia are not necessarily related to full loss of function. Rotatory tests are the standard for defining bilateral peripheral vestibular failure.^3^ Bilateral post-caloric arreflexia with absent rotatory test responses is associated with compromised bodily balance, which is more intense in cases where there is poor vision.^31^ Among the complaints that are reported, oscillopsia - the perception in which objects within the visual field appear to oscillate while walking - appears to be the most distressing.^41^ This condition results from loss of the VOR, which is necessary for stabilizing vision during movement.^4^, ^5^

Ototoxic drugs, such as gentamicin, may cause bilateral arreflexia.^34^ No cause is found in about 20% of bilateral arreflexia.^31^, ^41^

### Impaired suppression of nystagmus

Neurological diseases that inhibit the suppression of post-caloric nystagmus are commonly related with altered oculomotor movements (saccadic, tracking and optokinetic movements).^42^ If impaired suppression of nystagmus is bilateral, there is the possibility that the brainstem or the cerebellum is diffusely involved. In such cases, other neurological signs should be investigated, to confirm the diagnosis.^43^

### Caloric inversion

A caloric response in the opposite direction to the expected one is named caloric inversion.^17^ It is rare, and has been associated with brainstem disease.^44^ Also, technical errors may also cause caloric inversion, and should be investigated. The most common error is to place the electrodes incorrectly. Other possibilities are the presence of congenital nystagmus in the opposite direction to that expected from stimulation, and the presence of tympanic perforation when air stimulation is done.^29^

### Caloric perversion

Vertical nystagmus during caloric testing has been referred to as caloric perversion.^44^ This is a rare finding, associated with diseases that affect the floor of the fourth ventricle in the brainstem.^45^ An example is multiple sclerosis.^28^, ^45^

### Dysrhythmia of caloric nystagmus

This is defined as amplitude and frequency tracing irregularities. This finding has been associated with motor neuron diseases.^46^ Dysrhythmia of caloric nystagmus may also be seen in anxious or fatigued patients.^30^ The use of inadequate alert exercises or the presence of Bell's phenomenon may result in periodic loss of the caloric response.^47^

### Variables and artifacts

 

### Temperature

Temperature calibration should be as accurate as possible. For instance, in water stimulation, a 1°C temperature variation from the ideal 44°C or 33°C will result in a 14% difference in the stimulation magnitude.^12^

### Habituation

Repeated irrigation of the same ear with the same caloric stimulus results in a gradual decrease of the caloric response, leading to vestibular habituation.^48^

### State of alertness

The pattern of nystagmus may vary according to the state of alertness of a patient. Lack of attention or drowsiness may result in absent or intermittent caloric responses, which may be erroneously interpreted as decreased vestibular function.^31^, ^49^ Plain talk may be more effective for keeping patients alert without inducing anxiety than a series of questions on arithmetics.^49^

### Anxiety and nervousness

Studies have shown that 10% of subjects presenting hyperreflexia have no evidence of organic disease.^26^, ^30^ Anxiety about an unknown test is the commonly accepted explanation. Thus, orientation about the steps in caloric testing and the possibility of vertigo are important for patients.^30^

### Tympanic membrane alterations

Water stimulation is not indicated in patients with tympanic perforation.^29^ The alternative is air stimulation. Tympanic perforation, however, is a confounding factor when analyzing the results of air stimulation testing. The issue in these cases is whether vestibular function is present or absent. Vestibular system symmetry calculations are of no use, as stimulation will differ when comparing one side with the other. Furthermore, air irrigation in these cases may result in nystagmus where the caloric response is opposite the expected one.^29^ Theoretically, warm air would evaporate from the middle ear during stimulation, and the resulting humidity would cause a cooling effect, which might inhibit the caloric response instead of generating an excitatory response.^29^

### Lighting

Caloric testing is usually performed in one of three environmental situations:

1) eyes open in a completely darkened room;
2) eyes closed in a semi-darkened room; and
3) eyes open using Frenzel goggles in a semi-darkened room or using a video Frenzel. These goggles are connected to a video system, and exclude light completely while keeping the patient's eyes open; nystagmus is recorded by the video system.^50^ The best ambience for assessing vestibular function is a completely darkened room in which patients keep their eyes open.^2^, ^50^

Caloric testing in a semi-darkened room with the patient's eyes closed is also valid. Bell's phenomenon is an artifact that may be seen, however, if post-caloric nystagmus with the eyes closed is absent and if nystagmus occurs when the patient is asked to open the eyes for ocular fixation.^47^ Testing in this manner may be done if the results of caloric testing done with the eyes closed with those done with the eyes open in a completely darkened room or using a video Frenzel are compared.^50^, ^51^

### Bell's phenomenon

This is defined as ocular globe deviation and adduction that occurs in certain subjects upon closing the eyes, inhibiting post-caloric nystagmus. Consequently, the post-caloric response is absent in these normal subjects when their eyes are closed, and present when their eyes are open. This condition may mimic the absence of the inhibition effect of visual fixation, which is considered a sign of central injury.^50^, ^51^

### Drugs

Certain drugs that are used continuously may impair the control of oculomotor movements, and may invalidate or impair the interpretation of caloric testing results.^52^ Examples are antipsychotic, antidepressant and anticonvulsant drugs.^52^ Tracking and saccadic movements may be altered and spontaneous nystagmus may be present. Test analysis should always take into account the interference of drugs that patients may be using, as those alterations are associated with central nervous system diseases, and withholding the drug is not always possible prior to vestibular testing.^2^, ^42^, ^52^ Other conditions associated with drug use are suppression of the caloric response or loss of the ability to suppress post-caloric nystagmus by ocular fixation.^42^ Therefore, drugs that inhibit vestibular functions and that may be withheld abruptly with no harm to patients should be stopped 48 hours before caloric testing.^2^ These drugs include cinnarizine, flunarizine, dimenhydrinate and alcoholic beverages.^2^, ^51^

Moderate intake of caffeine and its derivatives (not more than 3 cups/24 hours) may be allowed, as long as the last cup is taken at least six hours before testing;5 the same applies to tea, chocolate and smoking. The reason for not stopping these substances 48 hours before testing is that sudden interruption could cause anxiety in patients, which alters test results.^26^, ^30^, ^53^ Furthermore, the half life of these substances is short, raging from 4 to 6 hours.^52^, ^53^

In summary, drugs that are used continuously for the treatment of neurological, psychiatric and cardiovascular conditions should not be stopped.^2^, ^52^

If altered test results are possibly related to drugs being taken, testing should be repeated after withholding those drugs and informing the patient's attending physician.^2^

### Position of the head / body

Coats and Smith (1967) carefully studied the relation between the caloric response and body position across 360°. The maximum response occurred when placing the head between 0° and 60° in the supine position for both warm and cold-water irrigation. In this method, deviations from the ideal inclination of the head have little effect on the caloric response.^54^

### Eye blinks

Eye blinks may interfere on the interpretation of caloric responses. The biphasic eye movement generated by blinking is shaped as an “acute peak” with no definition of a slow or rapid phase. This finding helps differentiate eye blinking from vestibular nystagmus.^2^, ^51^

### Overlap of congenital nystagmus

Congenital nystagmus may influence the interpretation of spontaneous, optokinetic, positional and positioning nystagmus, which in turn may make the interpretation of caloric testing more difficult.^1^, ^2^ Overlap of congenital nystagmus should be algebraically added or subtracted from evoked post-caloric nystagmus.^1^

### Monothermal caloric testing

Barber et al. (1971) first investigated the efficiency of using only warm caloric testing.^55^ This technique is limited by false-negative results.^51^ Using monothermal stimulation as a screening method may be indicated for patients presenting vague complaints, probably of non-vestibular origin, and in children with possible unilateral vestibular deficits.^55^ Monothermal caloric testing was developed only for these specific cases.^51^

### Ice caloric testing

This technique historically has been reserved for patients that do not respond to water caloric stimulation at 30 and 44°C. A qualitative approximation is made when assessing responses at lower temperatures; only the presence or absence of nystagmus and symmetry between irrigation of both sides are recorded.^2^ Ice caloric testing has been less used, as the definition of bilateral failure is usually established by rotatory testing.^3^, ^4^

## FINAL COMMENTS

This review shows that this theme has been widely investigated, and that there is conformity about what to expect from caloric testing. After many years of use for assessing vestibular function, caloric testing remains the main test for evaluating peripheral vestibular function. Modern technology has improved the test accuracy and sensitivity. It is thus important to be technically precise when undertaking caloric testing, being always aware of possible sources of error.
